# Editorial: Highlights in sports science, technology and engineering 2021/22

**DOI:** 10.3389/fspor.2022.1117803

**Published:** 2023-02-01

**Authors:** Peter Düking, Pietro Picerno, Valentina Camomilla, Laura Gastaldi, Billy Sperlich

**Affiliations:** ^1^ Integrative and Experimental Exercise Science, Department of Sport Science, University of Würzburg, Würzburg, Germany; ^2^ SMART Engineering Solutions & Technologies (SMARTEST) Research Center, Università Telematica “e-Campus”, Novedrate, CO, Italy; ^3^ Department of Movement, Human and Health Sciences, University of Rome Foro Italico, Rome, Italy; ^4^ Department of Mechanical and Aerospace Engineering, Politecnico di Torino, Torino. Italy

**Keywords:** wearable, sensor, markerless motion capture, muscle parameters, health monitoring, virtual reality, sport equipment

**Editorial on the Research Topic** Highlights in Sports Science, Technology and Engineering: 2021/22

## Introduction

Sports Technology and Engineering is a trending sub-category translating the innovative use of technology and engineering design into practice and encompasses many disciplines of sports science (e.g., sports medicine, prevention, rehabilitation, athletic development, etc.). Research in this field often aims to develop materials, sensors, algorithms, or full pieces of equipment to maintain and improve certain dimension of health, lifestyle and/or performance in different populations (e.g., able-bodied and disabled, sedentary, diseased, fitness oriented, competitive, or athletic). Such sports technologies are either developed from a research perspective to increase the understanding of operating principles and adaptation mechanisms (e.g., employing sensors and algorithms to monitor a variety of parameters in different settings) or from an applied perspective to provide technologies which optimize training, competition, or lifestyle activities.

**Figure 1:**
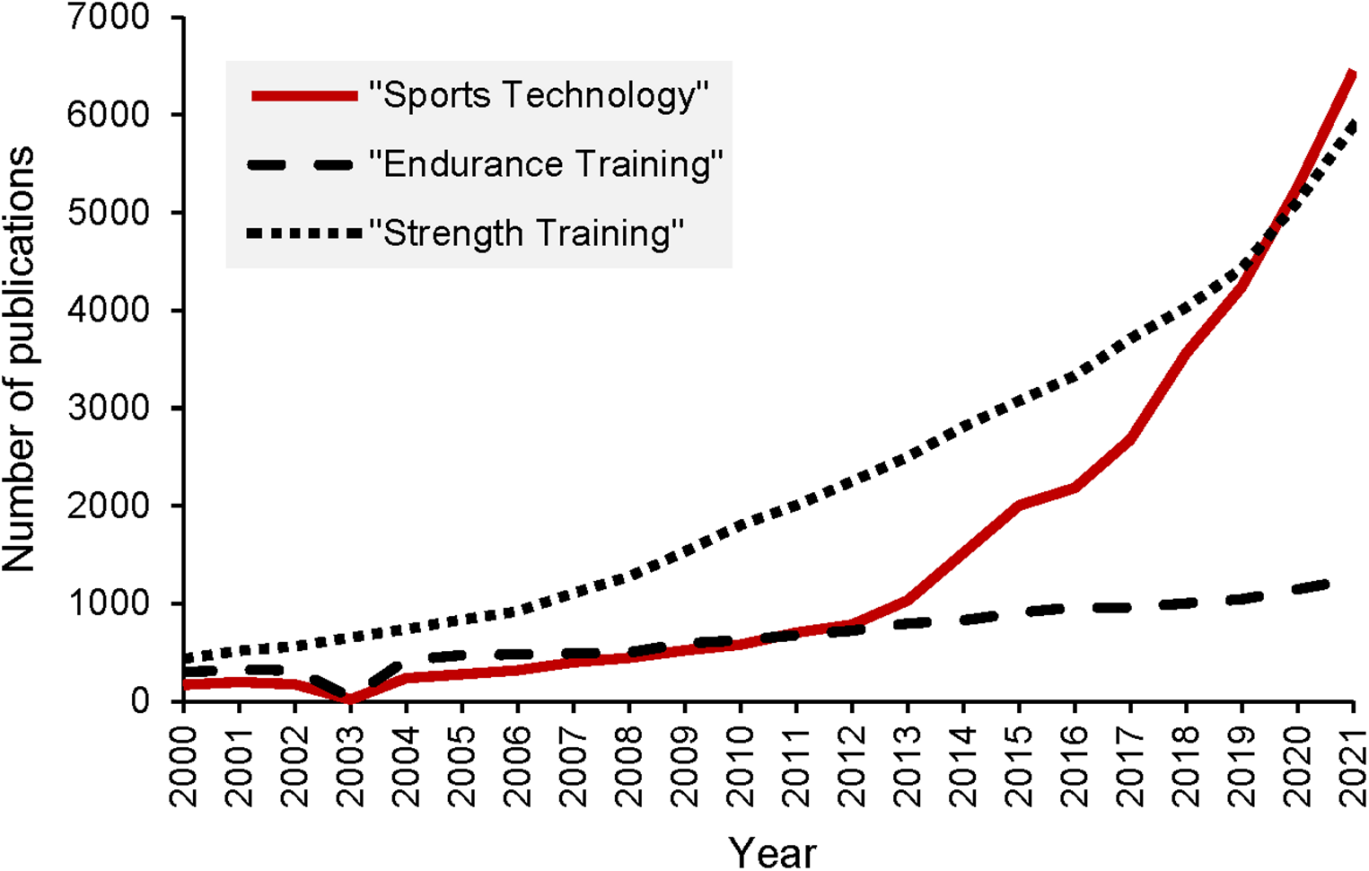
Number of publications for “Sports Technology”, “Endurance Training” and “Strength Training” in Pubmed.gov as of 07.11.2022.

Research around sports technology is growing rapidly and even outpaces other areas of interest in the field of sports science. Figure 1 illustrates the number of yearly publications listed in the Pubmed database. In 2000, 168 articles were published in Pubmed for the search-term “Sports Technology” and for example “Endurance Training” or “Strength training” hit 299 and 437 numbers of publications, respectively. While the numbers of all these search terms increased since 2011 year-by-year annual publications for “Sports Technology” (*n* = 702 in 2011) have surpassed those for “Endurance Training” (*n* = 678 in 2011). Since 2020, the annual number of publications for “Sports Technology” (*n* = 5,264 in 2020) is also higher than the yearly number of publications for “Strength Training” (*n* = 5,114 in 2020).

The variety of sports technology research is reflected in the present research topic including 10 articles from 54 authors out of which six are original research articles, one perspective article, one research report, one case report and one methodological article.

The aim of this research topic was to gather scientific contributions from the broad variety of research performed across the Sports Science, Technology and Engineering section in the years 2020–2021 to highlight the current main areas of interest, as well as emerging applications and trends. Here, the potential for sports technology and engineering research describes the potential to enhance “in the field” motion analysis, investigate mechanisms of adaptation, and enhance health monitoring and training in different populations and settings.

Two papers tested markerless motion capture for “in the field” sport motion analysis, through the assessment of upper and lower limbs kinematics. In this regard, Lahka and colleagues aimed at evaluating the concurrent validity of a bidimensional markerless motion capture system in assessing upper limbs kinematics of elite boxers while performing some typical in-ring boxing maneuvers Lahkar et al. while Pinheiro et al. tested the open-source OpenPose software for penalty kick analysis in elite football players from TV footage as compared to an observational analysis.

Two papers investigated technologies to assess muscle parameters during exercise: Puce et al. investigated the correlation between spectral parameters obtained from surface electromyography and variations of kinematic data and mechanical fatigue in elite swimmers. McPhail et al. and co-workers evaluated the within-session reliability of some force-related performance parameters during a novel unilateral isometric hex bar pull as performed by male and female elite freeski athletes on a force plate at the maximal voluntary contraction, while providing sex- and level specific reference values.

Health monitoring was the focus of three contributions: Ausland et al. proposed the use of a new mobile, long-term electrocardiogram (ECG) monitoring patch to assess automatic arrhythmia detection during endurance training in elite athletes. Bender et al. reported a device capable of automatically analyzing urine specific gravity (i.e., an index of hydration status of individuals) in real time. Finally, Fraysse et al. report physical activity cut-points for wrist-worn technologies in elderly populations (>70 years of age). Noteworthy, the authors investigate whether wear-side (i.e., dominant vs. non-dominant wrist) affects accuracy and conclude that wearing the technology on the dominant wrist might deliver more accurate data especially when individuals are active in low intensity zones. Schelling and co-workers presented a methodological framework for the design of a decision support systems for scheduling trips and training sessions in professional team sports.

Sports technology research contributes also to aspects of training, e.g., by augmenting the training environment using virtual reality, or adapting sport equipment to the specific athlete. In particular, the case report by Severin et al. and others compared the effects of adjusting seat and backrest angle on performance of an elite paralympic rower. In their perspective paper, McIlroy et al. shared their opinion concerning the use of virtual reality to train cyclists. The authors summarize strengths, weaknesses, as well as opportunities and threats of virtual online training platforms, with the attempt to enhance awareness of various aspects of virtual training technology and online cycling.

Throughout the reported research ideas and research outcomes, it remains clear that sports technology does not fulfill a purpose on its own, but it is a means to an end for sport scientists, applied practitioners, coaches and athletes. To increase our understanding of working principles and mechanisms, sports technology needs to be sound and reliable and must be flawlessly applied. In this regard, the field needs educated researchers and practitioners who understand how to employ and interact effectively and efficiently with technologies of different forms (1, 2). This interaction includes (i) having an evidence-based course of action on whether and when to apply technologies for a certain problem, (ii) the selection of appropriate technologies for a given purpose, population and setting, (iii) the appropriate management, handling and application of the technology and (iv) understanding of how to analyze and contextualize data correctly. Given the increasing availability of sports technologies, it seems necessary to constantly and critically evaluate new technologies and, at the same time, educate stakeholders in this area.
